# The Staphylococci Phages Family: An Overview

**DOI:** 10.3390/v4123316

**Published:** 2012-11-23

**Authors:** Marie Deghorain, Laurence Van Melderen

**Affiliations:** Laboratoire de Génétique et Physiologie Bactérienne, Faculté de Sciences, IBMM, Université Libre de Bruxelles (ULB), Gosselies, B-6141, Belgium

**Keywords:** bacteriophages, *Staphylococcus*, horizontal transfer, virulence

## Abstract

Due to their crucial role in pathogenesis and virulence, phages of *Staphylococcus aureus* have been extensively studied. Most of them encode and disseminate potent staphylococcal virulence factors. In addition, their movements contribute to the extraordinary versatility and adaptability of this prominent pathogen by improving genome plasticity. In addition to *S. aureus*, phages from coagulase-negative *Staphylococci* (CoNS) are gaining increasing interest. Some of these species, such as *S. epidermidis*, cause nosocomial infections and are therefore problematic for public health. This review provides an overview of the staphylococcal phages family extended to CoNS phages. At the morphological level, all these phages characterized so far belong to the *Caudovirales* order and are mainly temperate *Siphoviridae*. At the molecular level, comparative genomics revealed an extensive mosaicism, with genes organized into functional modules that are frequently exchanged between phages. Evolutionary relationships within this family, as well as with other families, have been highlighted. All these aspects are of crucial importance for our understanding of evolution and emergence of pathogens among bacterial species such as *Staphylococci*.

## 1. Introduction

The vast majority of bacteria contain prophages, either integrated into their chromosome or as extra-chromosomal elements, accounting for substantial genetic variability. Not only do phages shape bacterial genome architecture, they also constitute major vehicles for horizontal gene transfer [1,2]. In addition, they contribute to virulence by encoding numerous virulence or fitness factors and by their movements within genomes (see below, Section 2.2.) [2,3,4,5]. These mobile elements are responsible for gene disruption, provide anchor region for genomic rearrangements, protect bacteria from lytic infections or, in contrast, provoke cell lysis through prophage induction [2]. Thus, phages play essential roles in bacterial evolution and adaptation.

Phages are widespread in *Staphylococcus aureus* and have been extensively studied [1,3,6]. They were firstly used for the typing of clinical *S. aureus* isolates [7,8]. *S. aureus* is a major human and animal pathogen that causes both nosocomial and community-acquired infections. It colonizes skin and mucous membranes, with the anterior nares being the primary niche in humans. While found in healthy carriers, *S. aureus* is also responsible for a wide range of diseases, from mild skin infections to severe life-threatening infections, such as sepsis or endocarditis [6]. The number of prophages in *S. aureus* genome is generally high. All *S. aureus* genome sequenced so far do contain at least one prophage, and many strains carry up to four [1]. These encode numerous staphylococcal toxins responsible for pathogenesis [1,2,6].

*Staphylococci* also comprise coagulase-negative species (coagulase-negative *Staphylococci*, CoNS), which are distinguishable from *S. aureus* by the lack of coagulase-encoding gene. In contrast to *S. aureus*, which is only found in part of the population, these species belong to the commensal flora of healthy humans. Some species are associated to specific niches, and others appear to be more ‘generalist’ and are generally found on the body surface [9,10,11,12]. CoNS include human opportunistic pathogens often associated with medical devices. *S. epidermidis* is referred to as a frequent cause of nosocomial infections [9,10,11,12]. In addition, ‘true’ pathogens that are not associated with medical devices may also be problematic for public health. As an example, *S. saprophyticus* is considered as a frequent pathogen responsible for uncomplicated urinary tract infections [11,13]. Pathogenesis of CoNS species relies on factors required for their commensal mode of life or fitness (e.g. factors involved in adhesion, in biofilm formation and in persistence) and not on toxins, as observed for *S. aureus* [10,11]. As a consequence, less attention has been paid to these phages.

During the past decade, sequencing of *Staphylococci* genomes and extensive comparative genomic analyses have significantly increased the number of staphylococcal phages identified. Up to now, more than 68 *Staphylococci* phages and prophages sequences, mainly from *S. aureus*, are found in the [14]. In addition, 268 *Staphylococci* genomes are available on the PATRIC server [15] and offer a remarkable source of novel prophage sequences for further studies (see below).

In this review, we provide an overview of *Staphylococci* phages with a focus on their contribution to pathogenesis. A special interest is placed on the classification methods, as well as on the evolutionary relationships connecting staphylococcal phages. Phage classification is often problematic, due to the modular organization of phage genomes. Relationships between *Staphylococci* phages and phages from other species are also discussed in an evolutionary perspective. Finally, the potential use of staphylococcal phages for bio-technological and medical applications is briefly addressed.

## 2. The Phages of *S. aureus*

### 2.1. Global Features of *S. aureus* Phages

#### 2.1.1. Morphological Families

As the vast majority of phages, the *S. aureus* phages known so far are double-stranded DNA phages belonging to the *Siphoviridae* family of the *Caudovirales* order (Table S1) (reviewed in [2,4,6]). In general, they are temperate phages detected as prophage inserted in the chromosome, some of them being lytic due to mutations in the lysogeny functions (e.g., phiIPLA35 and phiIPLA88; [16]; or SA11; [17]). According to the morphological classification previously proposed by Ackermann [18], staphylococcal *Siphoviridae* are composed of an icosahedral capsid and a non-contractile tail ended by a base-plate structure. Capsids may adopt elongated or isometric shapes, and tail length varies from short (130 nm) to long (400 nm). A small number of *S. aureus Podoviridae* and *Myoviridae* phages, also belonging to the *Caudovirales* order, were described (Table S1). *Podoviridae,* such as the recently identified SAP-2 phage [19], are composed of a small icosahedral capsid and a short, non-flexible, non-contractile tail. *Myoviridae* phages, such as the well-known phage K [20], are characterized by an icosahedral capsid and a long contractile tail.

#### 2.1.2. Genomic Characteristics of *S. aureus* Phages

A comparative study of *S. aureus* phage genomes performed by Pelletier and co-workers [3] revealed several key genomic features, which are globally applicable to all staphylococcal phages described so far. The analysis encompassed 27 genomes from *S. aureus* phages and prophages belonging to the three morphological families described above.

Genome size extends from less than 20 kb up to more than 125 kb [3]. In contrast to phages from *Mycobacterium* [21] or *Pseudomonas aeruginosa* [22], genome sizes are not uniformly distributed, and three categories can be established and used to classify *Staphylococci* phages (class I: < 20 kb; class II: ≈ 40 kb, class III: >125 kb; see Section 4.1) [3]. Interestingly, genome size categories correlate with the morphological classification, *Podoviridae* harboring the smallest genomes (class I), *Myoviridae* the largest ones (class III), and *Siphoviridae* showing intermediate sizes (class II).

Coding regions are tightly packed with very few and small intergenic regions and a high gene density (1.67 genes/kb in average) [3]. Their GC content is similar to that of the host. *S. aureus* phages provide an impressive, mainly unexplored source of genetic diversity. On the 2,170 predicted proteins from the 27 phages analyzed in the study of Pelletier and co-workers, a function could be assigned using BLAST to 35% of the ORFs. No match was detected for 44% of these ORFS in Bacteria and Phages Gene Bank databases [3].

**Figure 1:**
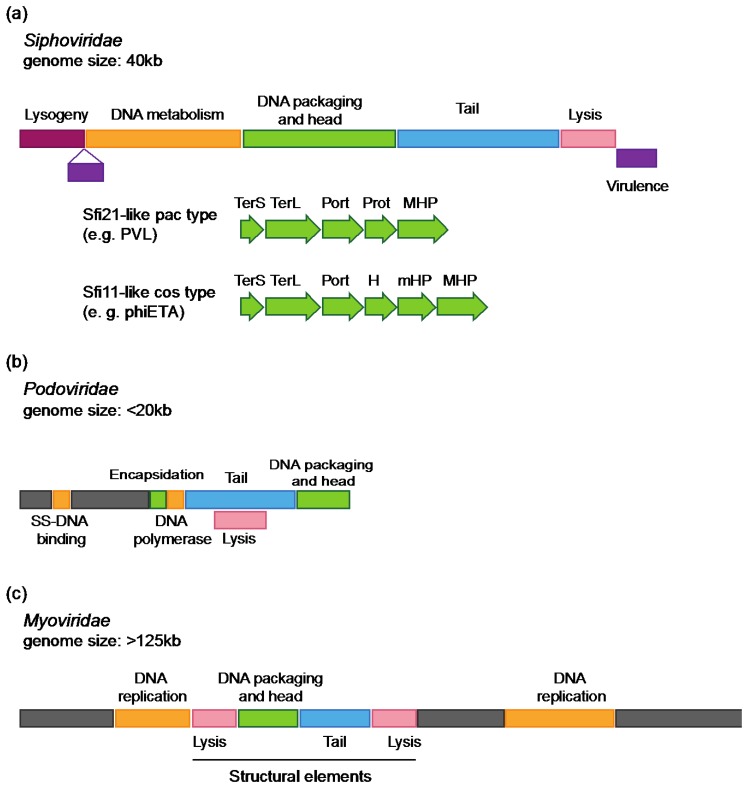
Modular organization of *Staphylococci* phages genomes **(a) ***Siphoviridae* genomes. Colored boxes represent the five functional modules found in *Siphoviridae* genomes. Red: lysogeny, yellow: DNA metabolism, green: DNA packaging and capsid morphogenesis, blue: tail morphogenesis, pink: cell host lysis. Virulence genes (purple) are generally found downstream the lysis module, or inserted between the lysogeny and DNA metabolism module. A closer view of the DNA packaging and capsid morphogenesis shows the structural genes pattern typical of the Sfi21- and Sfi11- like phages genera (see text for details); **(b)** *Podoviridae* genomes. Lysis module (pink) and tail morphogenesis modules (blue) are overlapping. DNA metabolism genes (*i.e.*, single-strand DNA binding protein and DNA polymerase; green) are located in a region of genes of unknown function (gray), upstream to the tail module. An encapsidation protein is encoded next to the DNA polymerase in the staphylococcal *Podoviridae* genomes described so far. **(c)** *Myoviridae* genomes. The phage Twort genome is represented as an example. A large region encodes genes of unknown function (gray). DNA metabolism genes (yellow) are distributed in two distinct modules (known as replication modules), as well as lysis genes (pink) that are found upstream and downstream to DNA packaging and capsid (green) and tail modules (blue). TerS: small subunit terminase, TerL: large subunit terminase, Port: portal protein, Prot: protease, MHP: major capsid protein, H: capsid morphogenesis protein, mHP: minor capsid protein, SS-DNA binding: single strand DNA binding protein.

Genomes of *S. aureus Siphoviridae* display the typical structure of the morphological family (Figure 1a) [2,4,23]. Five functional modules are arranged as follows: lysogeny, DNA metabolism, DNA packaging and capsid morphogenesis, tail morphogenesis and host cell lysis. The DNA metabolism module can be divided into replication and regulation functions. When present, virulence factors are generally encoded downstream of the lysis module [2,4]. In some cases, they are inserted between the lysogeny and DNA metabolism modules as reported for phiNM1 to four prophages found in the *S. aureus* Newman strain [24]. Genes are generally transcribed on the same strand, except for small clusters, such as genes involved in host genome integration [3,4].

While harboring a modular structure as well, organization of *Podoviridae* genomes is different (Figure 1b) [3,19,25]. One major distinction resides in a smaller number of ORFs, as indicated by a smaller size (20 to 32 ORFs) [3,19,25]. Functional modules encoding DNA packaging and capsid morphogenesis, tail morphogenesis and lysis were identified, in addition to genes of unknown function. In contrast to *Siphoviridae*, modules are not well defined and tail and lysis genes are overlapping. In addition, the lysogeny module is absent as expected for lytic phages.

Genome organization of staphylococcal *Myoviridae* (e.g. phages K, G1, Twort) [3,20,26] is similar to *E. coli* T4 phage, the paradigm for *Myoviridae* [27]. Genomes are organized into functional modules of conserved genes (replication, structural elements), interrupted by large plastic regions encoding mainly genes of unknown function (Figure 1c). Structural modules found in staphylococcal *Myoviridae* phages are more closely related (in terms of gene content and organization) to modules found in staphylococcal *Siphoviridae* phages than to modules of *Myoviridae* found in different bacterial species.

### 2.2. Role of Phages in *S. aureus* Pathogenesis

The *S. aureus* genome is mainly composed of a backbone of genes that are conserved among the different strains, both in terms of sequence and synteny [28,29]. These genes constitute the ‘core genome’ in opposition to the ‘accessory genome’, which is variable between strains and constituted by integrated plasmids, transposons, genomic islands, pathogenicity islands (saPIs) and prophages. This ‘accessory genome’ may represent up to 25% of *S. aureus* genome and largely contributes to the high genetic and phenotypic plasticity of the pathogen. Indeed, one of the remarkable characteristics of *S. aureus* is represented by its versatility and ability to adapt to diverse and hostile environments [1,6,28,30,31], and phages are playing an essential role in this phenomenon.

#### 2.2.1. Phages-encoded Virulence Factors

Phages encode a large proportion of *S. aureus* virulence factors and provide the pathogen with a large variety of toxins, mainly allowing escaping host immune system (reviewed in [1,6,31]). Many factors have been described and characterized, such as the widespread immune modulator staphylokinase (*sak*) responsible for host tissue destruction, the chemotaxis inhibitory protein CHIP (*chp*), the staphylococcal inhibitor of complement SCIN (*scn*) and several superantigens (*sea*, *seg*, *sek*, *sek2*, *sep*, *seq*). These superantigens are enterotoxins causing food poisoning, toxic shock syndrome and necrotizing fasciitis. In addition, the bi-component cytotoxin Panton-Valentine leukocidin (PVL, encoded by *lukF-PV*, *lukS-PV*) and related leukocidins (*lukM*, *lukF*)) form pores into leukocytes and cause necrotic infections. Finally, the exfoliative toxin A (*eta*) is involved in severe skin infections.

In general, phages are carrying single virulence factor genes, although some exceptions have been reported. For examples, phiSa3 phages and relatives, such as phiN315, may encode up to five virulence factors, which form an immune escape complex (IEC) [5,31,32,33]. Virulence factor genes are not strictly associated to a specific phage and appear to be exchanged by horizontal gene transfer and recombination [2,6,31] (see Section 2.2.3). As mentioned above, virulence genes are often located near the attachment site (*att*) of the prophage, *i.e.*, adjacent to the host chromosome. A possible origin for their acquisition by phages might be aberrant excision events from an ancestral bacterial chromosome [34]. They might also derive from mobile genetic elements, as suggested by the presence of transposase genes flanking toxin genes as in phiPV83 [35]. Thus, phages clearly impact virulence by positive lysogenic conversion, since they provide novel functions and activities to the host. Negative conversion also occurs as prophage insertion might inactivate genes [2,4,31]. In most of the cases, both phenomena occur simultaneously. As an example, phiSa3-related phages integrate in beta-hemolysin gene, rendering lysogens defective for beta-hemolyse but effective for IEC production. The integration site relies on the specificity of the phage integrase [36,37].

Expression of phage-encoded virulence genes is maximal upon entry in the lytic cycle, since latent promoters are activated and phage genome is replicated, leading to an increase in genome copy number [5,38,39], although some expression is detected during lysogeny [34].

#### 2.2.2. Phage-mediated Mobilization of Virulence Factors: SaPIs Pathogenicity Islands

Phages are the primary vehicles for horizontal transfer between *S. aureus* strains. They spread chromosomally-encoded virulence determinants through generalized transduction. In addition, they are also responsible for the mobilization of SaPIs, which encodes major toxin genes, such as the toxic shock syndrome toxin 1 and other superantigens (reviewed in [40]). SaPIs are widespread in *S. aureus* genomes. They are discrete chromosomal DNA segments that have been acquired by horizontal transfer. They are not mobile by themselves and rely on a helper phage for moving. SaPIs are replicated and mobilized either in response to SOS-induced excision of a helper prophage present in the same strain, either following the infection by a helper phage or by the joint entry of SaPI and helper phage [40]. The underlying molecular mechanism for induction is the specific interaction of a SaPI repressor and a de-repressor encoded by the helper phage. Different proteins of a particular helper phage may be involved in induction of different SaPIs. In particular, phi80alpha is able to mobilize at least five different SaPIs [40,41,42]. Hence, SaPIs mobilization represents a remarkable example of evolutionary adaptation involving pathogenicity islands and phages [42].

#### 2.2.3. Phage Dynamics Contribute to *S. aureus* Evolution and Pathogenesis

Additionally to gene transfer, phages contribute to genetic alterations during infection, providing the species with broad genetic variations [5,38,43,44,45]. In addition to phage acquisition or excision (lysogenic conversion); duplication, ectopic integration and stable extra-chromosomal form of phages have been reported, increasing genetic diversity within bacterial populations [36,44]. Hence, generating heterogeneity within a population upon infection offers different virulence potentials and provides the pathogen with the ability to develop a flexible response to host defenses. It was shown that phage mobilization and atypical genomic integration are favored in pathogenic strains upon infection conditions, compared to colonizing strains in healthy carriers [5,36,44]. Factors causing phage induction *in situ* are environmental conditions that lead to bacterial DNA damage, such as antibiotics treatments or reactive oxygen species released by macrophages. Phage-mediated phenotypic diversification acts in concert with other mechanisms for genetic variations, such as recombination and mutations, and is under the influence of external factors, such as the presence of co-infecting species [43,45].

## 3. What About Phages in Non-*aureus Staphylococci*?

Comparison of CoNS genomes with *S. aureus* genomes revealed the inter-species conservation of both sequence and synteny of a large proportion of genes (core genes) with variable regions carrying species-specific genes [13,46,47,48,49]. Phage-encoded virulence factors responsible for *S. aureus* pathogenesis are absent in CoNS, in correlation with the difference in pathogenesis-mediated by CoNS. However, toxin and antibiotic resistance genes have been identified in several of these species. Although mobilization of virulence genes by phages has not been demonstrated, it is conceivable that phages might play a role in pathogenesis and evolution of CoNS, such as observed for *S. aureus*. Accordingly, phages were shown to impact genetic variability in *S. epidermidis* [50]. Although phage prevalence in clinical isolates might be underestimated [51], CoNS genomes described so far contain only few prophages or genomic islands, if any.

Most of the CoNS phages belong to the *Siphoviridae* family, while several virulent *Podoviridae* phages have been recently isolated directly from human anterior nares [52]. As for *S. aureus* phages, the first interest brought to phage infecting these species relied on their use for clinical isolate typing [53,54]. Several studies report the isolation and characterization of phages from *S. epidermidis* and *S. saprophyticus* [52,55,56,57], but only nine phages and prophages have been sequenced and studied, both at the molecular and physiological levels (Table S2). Among these, five phages and prophages are from *S. epidermidis* [51,58,59], one prophage from *S. carnosus* [48], two phages from *S. hominis* [37] and one phage from *S. capitis* [37]. The *S. epidermidis* vB_SepiS_phiIPLA5 (Table S2) is strictly lytic due to a defective lysogeny module [51,57], and the *S. carnosus* phiTM300 prophage (Table S2) appears to have lost its mobility, as it could not be induced upon mitomycin C treatment [48]. These CoNS phages show the general genomic features described for the *S. aureus* phages (see above, Section 2.1.2; Table S2). Their genome size range falls into the class II proposed by the Pelletier group [3], with high gene density and a typical genomic organization into five functional modules (lysogeny, DNA metabolism, DNA packaging and capsid morphogenesis, tail morphogenesis and lysis). As for *S. aureus* phages, a function could be only assigned to a small proportion of the ORFs (from 29% to 53%, depending on the phage). Known virulence determinants were not found in these genomes. Comparative analysis of genome sequences revealed that *S. epidermidis* vB_SepiS_phiIPLA5, vB_SepiS_phiIPLA7, phiPH15 and phiCNPH82 are closely related, while phi909 showed a high similarity to *S. aureus* phages [48,51]. Interestingly, we also detected close relationships between *S. aureus* and CoNS phages (see below, Section 4) [37].

Genomic islands related to SaPIs elements were identified in *S. haemolyticus* [46] and *S. saprophyticus* [13], although they usually lack superantigen-encoding genes. The first CoNS superantigen-bearing genomic island was recently described in *S. epidermidis* [59]. SePI-I encodes the staphylococcal enterotoxin C3 (SEC3) and enterotoxin-like toxin L (SEIL). Interestingly, the *seil* gene is homologous to those of *S. aureus*, indicating horizontal transfer events between *Staphylococci* species.

## 4. Classification and Evolution of the Staphylococcal Phage Family

### 4.1. Classification of Staphylococci Phages, a Long-Term Challenge

Early classifications proposed for *S. aureus* phages were based on their lytic properties, serotypes, morphology, on number and size of virion proteins and on genome size and organization as revealed through DNA hybridization or endonuclease restriction patterns ([18,60,61,62,63]; reviewed in [26]). More recently, progress in genomics and bio-informatics have allowed alternative classification methods.

Comparative genomic studies led to the subdivision of the morphological families into sub-families and genera. Following a classification proposed by Brussow and Desiere (although still not recognized by ICTV (International Comity on Taxonomy of Viruses)), *Siphoviridae* phages of low GC gram-positive bacteria, including *Staphylococci,* are categorized as Sfi21- or Sfi11-like phages by several authors [64] (Table S1 and Table S2). This distinction is based on the capsid genes pattern as reported for *Streptococcus thermophilus* phages [65]. Sfi21-like phages share characteristic features over the capsid region with *E. coli* HK97 phage and use a similar cos-site based strategy for DNA packaging (Figure 1a). In *S. aureus*, Sfi21-like genus is subdivided into three groups: the first being represented by phiPVL-phiPV83-phi13-phiSa3mw phages, the second by phiSLT-phiSa2mw-phi12 and the third by phiMu50A-phiN315 [4] (Table S1). While showing different capsid morphology (isometric or elongated), these phages encode typical capsid gene pattern of Sfi21-like phages (portal protein-protease-major capsid protein). Sfi11-like *pac*-type phages are related to the *B. subtilis* SPP1 phage and differ from Sfi21-like phages, notably by the lack of the protease-encoding gene (Figure 1a) [64,65].

The two other families of the *Caudovirales* order were recently reassessed on the basis of protein similarities [66,67]. Among *Podoviridae*, *S. aureus* phages constitute a novel genus called 44AHJD-like. This genus belongs to the *Picovirinae* sub-family that also includes the phi29-like genus represented by the well-described *Bacillus* phi29 [67]. Among *Myoviridae*, the Twort phage is a representative of the Twort-like genus within the *Spounaviridae* sub-family [66]. This sub-family also includes the *Bacillus subtilis* SPO1 phage and relatives, as well as the *Lactobacillus plantarum* LP65 phage [66,68].

Other classification approaches based on specific marker genes found within *S. aureus Siphoviridae* genomes have been proposed [29,63,69,70]. They rely on PCR detection of genes representative of different phage types or categories. In addition to providing classification schemes, these methods are useful for *S. aureus* prophage detection, which is of great interest for epidemiological studies. The markers encompass genes coding for structural components, such as tail fibers, capsid proteins [63] or integrase genes [29,36]. In the latter case, classification correlates with distinct integration sites into the host chromosome. A good correlation between the type of integrase and virulence determinants has also been reported [36]. However, these methods do not provide information about mosaic structure, although detection of representatives of each functional module might provide some clues about mosaicism [70].

**Figure 2:**
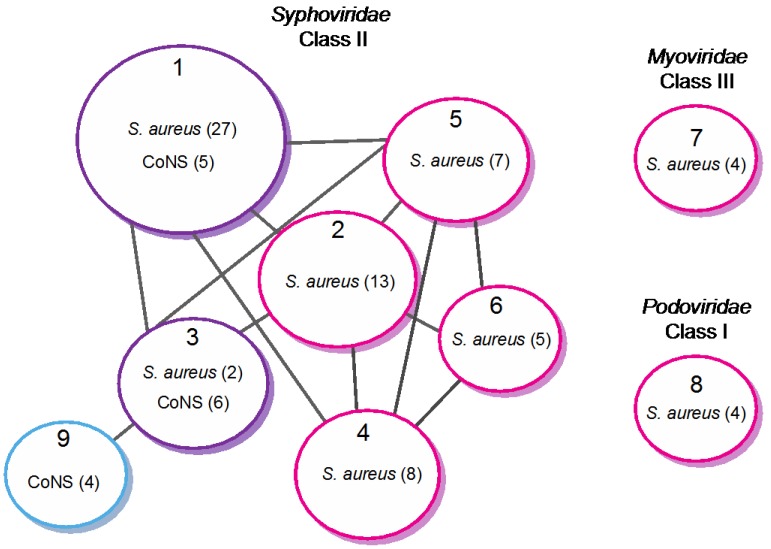
Network representation of relationships between *Staphylococci* phages based on protein content (adapted from [37]). Circles represent the nine different clusters defined by Markov cluster algorithm (MCL). The color indicates the host species (magenta: *S. aureus*; purple: *S. aureus* and CoNS; blue: CoNS). The number of genomes is indicated into brackets. Cluster 8 corresponds to the class I (*Podoviridae*), clusters 1–6 and 9 to class II (*Siphoviridae*) and cluster 7 to class III (*Myoviridae*). Cluster 1 corresponds to clade A, cluster 4 to clade B, and clades C and D were split into two sub-clades (clusters 2 and 3, and 5 and 6, respectively). Cluster 9 constitutes a new clade. In this schematic representation, gray lines between distinct clusters indicate that at least 30% of homologous proteins are shared between at least two phage genomes. Following this analysis, two previously unclassified phages (2638A and 187) were included in cluster 6 and 1, respectively. PT1028 was not included in this analysis.

Recently, the group of Pelletier [3] proposed a classification taking into account the genome size, the gene organization, in addition to comparative nucleotide and protein sequence analysis. Using comparative genomic, Class II (*Siphoviridae*) was divided into three clades (A-C), and a new clade (D) was added later on by the Fischetti group after including *S. epidermidis* and additional *S. aureus* phage sequences [58]. Our group has refined this classification by extending the analysis to 85 phage and prophage genomes, among which 15 originated from CoNS [37]. The approach was based on the similarity of protein repertoires using tree-like and network-like methods [71,72]. Both methods established nine distinct clusters (data obtained with the network-like method is shown in Figure 2). Seven of these clusters (1–6, 9) are composed of *Siphoviridae* and constitute class II, according to the Pelletier classification. Clusters 7 and 8 constitute class III and I and are composed of *Myoviridae* and *Podoviridae*, respectively. They are unrelated to the other clusters. Within class II, our analysis generates seven related clusters, instead of the four clades previously proposed by the Pelletier group. Most importantly, one cluster (cluster 9) is composed exclusively of non-*S. aureus* phages and constitutes an entirely new clade, as compared to the earlier classification. Four of the seven clusters are composed exclusively of *S. aureus* phages (clusters 2, 4, 5 and 6). Interestingly, the two last clusters (clusters 1 and 3) are composed of *S. aureus* and non-*S. aureus* phages, revealing close relationships between phages of different *Staphylococci* species.

### 4.2. Modular Evolution of Staphylococci Phages

#### 4.2.1. Extensive Genome Mosaicism in *Staphylococci* Phages

The mosaic gene organization is consistent with the theory of modular evolution based on module exchanges by horizontal transfer and recombination events [3,4,73]. This mosaicism can be viewed at either the nucleotide or amino acid level. At the nucleotide level, genome comparisons reveal exchange events that are likely to have occurred recently. Comparisons at the protein level identify homologous proteins that are shared by distantly related phages. They clearly derived from a common ancestor, but have diverged with time, and similarity at the nucleotide level is undetectable [73]. *S. aureus* phages are highly mosaic, indicating that gene exchange is common within this phage group [3,33,35,37,74,75,76,77,78,79]. Exchanges not only concern single genes, but also protein domains or a group of genes, such as functional modules [3,73]. Interestingly, *S. aureus* phages often share large, highly similar sequences with at least two other phages and with different phages along their genome. As an example, more than 80% of the ROSA genome is covered by large identical sequences from at least five other *S. aureus* phage genomes, with remarkable co-linearity. Exchange events are favored between phages of the same genome size range, which correspond to different morphological families (see above Section 2.1.2) [79].

Different molecular mechanisms have been proposed to explain how these exchanges occur [23,73]. A first model invokes homologous recombination events between conserved sequences, which are generally found at gene borders, such as detected in lambdoid *E. coli* phages [80,81]. Recombination events require large homologous regions that are recognized either by host- or phage-encoded recombinases [80]. A second model proposes alternative recombination mechanisms, occurring randomly or between short sequences [82]. It is likely that these two mechanisms contribute to mosaicism generation. For instance, PVL-related phage genomes are composed of long regions that are shared between different phages, flanked by conserved sequences, called junctions, suitable for homologous recombination events. However, shuffling might occur at other regions, since short regions appear to be shared by phage genomes [35,74,75,76]. Even though these mechanisms certainly lead to a high percentage of non-viable phages, it is considered as a highly creative process that might provide countermeasures against anti-phage mechanisms evolved by bacteria, such as CRISPR sequences, toxin-antitoxin systems and restriction-modification [82].

#### 4.2.2. Evolutionary Relationships within *Staphylococci* Phages

An interesting aspect of mosaicism is that comparison of a large number of genomes allows the establishment of phylogenetic relationships of specific regions instead of entire phages. Phylogenetic trees of representative genes revealed distinct evolutionary histories for different modules, thereby highlighting extensive mosaicism. This observation thus provides a tool to investigate evolution of *S. aureus* phages.

General clustering approaches, as used by our group to extend the classification of *Staphylococci* phages to CoNS species [37], are also useful to represent relationships among phage groups. In order to study the modular evolution of the CoNS StB12, StB27 and StB20 phages, families of homologous proteins were defined and the relative proportion of homologs in the nine clusters shown in Figure 2 was calculated. As previously shown [79], phages belonging to different morphological families do not share homologs. Our study confirms that morphogenesis module genes are in general less prone to horizontal swapping [3,73], although genes involved in host-phage interactions are notable exceptions. Clustering of *Staphylococcus Siphoviridae* phages relies mainly on structural features, indicating that within the overall similarities of virion structure, subtle variations allow differentiation. This is in accordance with a modular mechanism of evolution in which differentiation processes mainly rely on the exchange of a restricted number of genes or protein domains within structural modules [37]. Importantly, mosaicism encompasses genomes from both *Siphoviridae* phages from *S. aureus* and CoNS species, suggesting inter-species gene exchanges, which might be of crucial importance for *Staphylococci* pathogenesis.

### 4.3. Evolutionary Relationships between Staphylococci Phages and Other Species

Among the numerous approaches developed to outline evolutionary and functional relationships between phages [58,66,67,71,79,83,84,85,86,87], an original method developed by Lima-Mendez et al., [72,87] allowed them to study phage modular evolution on a large scale. The analysis was based on the clustering of phage proteins families in evolutionary conserved modules (ECMs) and the establishment of their distribution among phages. ECMs are defined as groups of protein that have a similar phylogenetic profile, meaning that they co-occur in genomes. In virulent phages, they are generally larger than functional modules, while in temperate phages, they tend to correspond to functional modules [87]. The clustering method allowed classification of phages into groups distinguished by different combination of ECMs. On the other hand, ECMs specific to particular phage groups can be identified. As an example, some modules were strictly associated with *Staphylococci* phages [72,87]. These ECMs encode virulence determinants, capsid and tail morphogenesis genes, or genes of unknown functions. The latter case is of special interest, since these ORFs of unknown functions might potentially be involved in phage/host interaction and may therefore include novel virulence factors or other proteins of medical relevance [72]. Other ECMs associated with *Staphylococci* phages were shared with *Streptococci* phages (modules involved in capsid and long non-contractile tail morphogenesis, phage integration/excision, replication or regulation functions), indicating an evolutionary link between these species.

Development of such methods is helpful for understanding phage evolution in general. These studies also complete the ICTV classification system based on morphology and specific molecular markers.

## 5. Use of *Staphylococci* Phages for Phage Therapy and Other Bio-Technological Applications

Due to the renewed interest for phage therapy, an increasing number of phages have been isolated and characterized for their potential use against *Staphylococci* infections, both in humans and animals (see notably [26,51,88,89,90,91]). Besides phage therapy, staphylococcal phages are also attractive candidates for food preservation [92,93,94,95]. Most phages selected for phage therapy or food preservation are strictly lytic because of the complications caused by lysogeny (e. g., resistance of lysogen strains to phage infection and unexpected transduction of host genes) [2,88,89]. The direct use of lysins is often an alternative for the use of entire phages (see notably [19,96,97]). 

Phage candidates are mainly virulent phages belonging to *Myoviridae* (e. g., phage K, [97,98] or the recently identified phiStau2A, [99]) and *Podoviridae* (e. g., phiSAP-2, [19]). They were isolated from diverse environments, including dairy products, farm environments (milk from cow infected mastitis), humans and medical devices implanted into patients (see notably [16,19,100]). 

Another important characteristic of phages selected for therapeutic and bio-technological applications is their narrow host range [89]. As an example, phage K appears to be specific to particular *S. aureus* clinical isolates, as well as to particular CoNS strains, while phiStau2 shows a larger host range among *S. aureus* clinical isolates, but is inefficient against the CoNS strains tested [99]. Therefore, to set up an efficient treatment, it is mandatory to precisely determine the bacterial species/isolate responsible for the infection, as well as disposing of a battery of phages of known host range. Traditionally, phage cocktails are used to overcome these issues [89,101]. On the other hand, this property allows specific treatment against pathogens without affecting the commensal flora. 

Nevertheless, phage therapy remains promising as a large number of phages—more than 10^31^ phage particles are estimated in the biosphere—await discovery, opening the perspectives for novel therapeutic approaches [89].

## 6. Concluding Remarks

The large number of *Staphylococci* phages sequenced and characterized so far revealed an extensive mosaicism, which indicates gene shuffling between phages of *Staphylococci* species, including *S. aureus* and CoNS. An important aspect of *Staphylococci* phages is their pivotal role in *S. aureus* pathogenesis. Horizontal transfer of phages is an efficient way to rapidly disseminate virulence determinants among pathogens. Such transfers have been reported between *S. aureus* clinical isolates during infections [6]. CoNS pathogenesis is less understood, and virulence genes appear to be absent in CoNS phage genomes. However, CoNS phage diversity remains to be explored, and their function in CoNS pathogenesis is likely to be underestimated. Identification and characterization of novel phages from pathogenic and non-pathogenic CoNS strains is therefore of crucial importance to further understand the evolutionary relationships connecting *S. aureus* phages with phages found in non-pathogenic commensal *Staphylococci*.

## Supplementary Files

Supplementary File 1
