# Supplementary material for: The Staphylococci Phages Family: An Overview
Source: Viruses. 2012 Nov 23;4(12):3316–35. doi: 10.3390/v4123316 (PMC3528268; doi:10.3390/v4123316)
Supplement: Supplementary File 1 [file viruses-04-03316-s001.pdf]

Table S1. *S. aureus phages*.

(Pro)phage	Genome size (pb)	Morphological		Cluster (Class)[g]	Genome sequence	Reference
		sub-family	genus			
Siphoviridae						
phi187	39,620	lambda supergroup	Sfi11-like[a]	1(II)	yes	[1]
phi69	42,732	lambda supergroup	Sfi11-like[a]	1(II)	yes	[1]
phi53	43,883	lambda supergroup	Sfi11-like[a]	1(II)	yes	[1]
phi85	44,238	lambda supergroup	Sfi11-like[a]	1(II)	yes	[1]
phi2638A	41,318	lambda supergroup	Sfi21-like[a]	6(II)	yes	[1]
phi77	41,708	lambda supergroup	Sfi21-like[a]	6(II)	yes	[1]
phi42e	45,861	lambda supergroup	Sfi21-like[a]	4(II)	yes	[1]
phi3A	43,095	lambda supergroup	Sfi21-like[a]	4(II)	yes	[1]
phi47	44,777	lambda supergroup	Sfi21-like[a]	4(II)	yes	[1]
phi37	43,681	lambda supergroup	Sfi11-like[a]	3(II)	yes	[1]
phiEW	45,286	lambda supergroup	Sfi11-like[a]	3(II)	yes	[1]
phi96	43,576	lambda supergroup	Sfi11-like[a]	2(II)	yes	[1]
phiROSA	43,155	lambda supergroup	Sfi11-like[a]	2(II)	yes	[1]
phi71	43,114	lambda supergroup	Sfi11-like[a]	2(II)	yes	[1]
phi55	41,902	lambda supergroup	Sfi11-like[a]	2(II)	yes	[1]
phi29	42,802	lambda supergroup	Sfi11-like[a]	2(II)	yes	[1]
phi52A	41,690	lambda supergroup	Sfi11-like[a]	2(II)	yes	[1]
phi88	43,231	lambda supergroup	Sfi11-like[a]	2(II)	yes	[1]
phiX2	43,440	lambda supergroup	Sfi11-like[a]	2(II)	yes	[1]
phi92	42,431	lambda supergroup	Sfi11-like[a]	2(II)	yes	[1]
phiPVL	41,401	lambda supergroup	Sfi21-like (gr1)[b,c]	5(II)	yes	[2, 3]
phiSLT	42,942	lambda supergroup	Sfi21-like (gr2)[b,c]	4(II)	yes	[3, 4]
phiPV83-pro	45,636	lambda supergroup	Sfi21-like (gr1)[b,c]	5(II)	yes	[3, 5]
phi108-PVL	44,107	lambda supergroup	Sfi21-like (gr1)[b,c]	5(II)	yes	[3, 6]
phiSa2mw	45,924	lambda supergroup	Sfi21-like (gr2)[b,c]	ND	yes	[6, 7]
phiSa2958	46,046	lambda supergroup	Sfi21-like (gr2)[b,c]	4(II)	yes	[3, 8]
phiSa2usa	43,062	lambda supergroup	Sfi21-like (gr2)[b,c]	ND	yes	[3, 9]
phi7247PVL	42,142	lambda supergroup	Sfi21-like (gr3)[b,c]	ND	yes	[3]
phi5967PVL	42,142	lambda supergroup	Sfi21-like (gr3)[b,c]	ND	yes	[3]
phiETA	43,081	lambda supergroup	Sfi11-like[b]	2(II)	yes	[10]
phiN315	44,257	lambda supergroup	Sfi21-like (gr3)[b,c]	6(II)	yes	[11, 12]

phiMu50A	43,053	lambda supergroup	Sfi21-like (gr3)[b,c]	ND	yes	[11, 12]
phiMu50B	44,391	lambda supergroup	Sfi11-like[b]	ND	yes	[11, 12]
phiSa3mw	ND	lambda supergroup	Sfi21-like (gr1)[b,c]	ND	yes (MW2 strain genome)	[7, 12]
phiSa3ms	42,612	lambda supergroup	Sfi21-like[d]	ND	yes	[13]
phiSa3	ND	lambda supergroup	Sfi21-like[a]	ND	yes (NCTC8325 strain genome)	[14, 15]
phi11	43,658	lambda supergroup	Sfi11-like[e]	1(II)	yes	[16]
phi12	44,970	lambda supergroup	Sfi11-like[e]	4(II)	yes	[16]
phi13	42,774	lambda supergroup	Sfi21-like[e]	5(II)	yes	[16]
L54a[f]	ND	ND	ND	ND	No	[17]
phiNM1	43,128	lambda supergroup	ND	1(II)	yes	[18]
phiNM2	43,145	lambda supergroup	ND	ND	yes	[18]
phiNM3	44,061	lambda supergroup	ND	6(II)	yes	[18]
phiNM4	43,189	lambda supergroup	ND	2(II)	yes	[18]
phi80	42,140	lambda supergroup	Sfi11-like[b]	2(II)	yes	[19]
phi80alpha	43,864	lambda supergroup	Sfi11-like[b]	1(II)	yes	[19]
phiMR11	43,011	lambda supergroup	Sfi11-like[a]	2(II)	yes	[20]
phiMR25	44,342	lambda supergroup	Sfi11-like[a]	ND	yes	[21]
phiIPLA88	42,526	lambda supergroup	Sfi11-like[b]	1(II)	yes	[22]
phiIPLA35	45,344	lambda supergroup	Sfi21-like[b]	4(II)	yes	[22]
TEM126	33,540	ND	Sfi11-like[b]	ND	yes	[23]
SA11	136,326	ND	ND	ND	yes	[24]
Podoviridae						
66	18,199	*Picovirinae*	44AHJD-like	8(I)	yes	[1]
44AHJD	16,668	*Picovirinae*	44AHJD-like	8(I)	yes	[1, 25]
P68	18,221	*Picovirinae*	44AHJD-like	8(I)	yes	[1, 25]
SAP2	17,938	*Picovirinae*	44AHJD-like	8(I)	yes	[26]
Myoviridae						
K	127,395	Spounaviridae	Twort-like	7(III)	yes	[1, 27]
G1	138,715	Spounaviridae	Twort-like	7(III)	yes	[1]
Twort	130,706	Spounaviridae	Twort-like	7(III)	yes	[1]
SAP1	ND	ND	ND	ND	No	[28]
SAP3	ND	ND	ND	ND	No	[28]
MSA6	140,194	Spounaviridae	Twort-like	ND	yes	[29, 30]
phi812	146,500	ND	ND	ND	No	[31]

SK311	141,100	ND	ND	ND	No	[31]
Stau2	134,500	ND	ND	ND	No	[32]
phiSA012	ND	ND	ND	ND	No	[31]
phiSA039	ND	ND	ND	ND	No	[31]
GH15	139,806	ND	ND	ND	yes	[33]
A5W	137,087	Spounaviridae	Twort-like	ND	yes	[30]
Staph1N	137,192	Spounaviridae	Twort-like	ND	yes	[30]
Fi200W	140,079	Spounaviridae	Twort-like	ND	yes	[30]
P4W	139,173	Spounaviridae	Twort-like	ND	yes	[30]
676Z	140,115	Spounaviridae	Twort-like	ND	yes	[30]
A3R	132,712	Spounaviridae	Twort-like	ND	yes	[30]
ISP	138,339	Spounaviridae	Twort-like	ND	yes	[30, 34]
Sb-1	127,188	Spounaviridae	Twort-like	7(III)	yes	[30, 35]
Unclassified						
PT1028	15,603	ND	ND	ND	yes	[1]

Prophages identified by genome strain sequencing and not further characterized are not indicated.

(a) Based on the presence of a putative prohead protease encoded within the DNA packaging and head morphogenesis module, not experimentally tested. Determined in this study

(b) Based on Authors information, experimentally tested or determined on the basis of head genes pattern

(c) Groups 1-3 within Sfi21-like genus determined on the basis of sequences comparison

(d) Determined in this study, based on the presence of a putative prohead protease encoded within the DNA packaging and head morphogenesis module, not experimentally tested. The phiSa3ms phage was indicated as a Sfi11-like *pac*-type phage by the Authors [13]

(e) Experimentally shown as Sfi11-like *pac*-type phage, although a gene encoding a putative prohead protease was annotated, which indicates Sfi21-like *cos*-type phage

(f) The L54a phage is likely a *Siphoviridae* phage since L54a and phi11 were shown to be closely related phages [16]

(g) Following the classification proposed by [40]

ND: not determined; the table is likely not exhaustive.

Table S2. *Staphylococci* phages from non-*S. aureus* species

(Pro)phage	Genome size (pb)	Morphological			Cluster (Class)[c]	Genome sequence	Host	Ref
		family	sub-family	genus				
CNPH82	43,420	*Sipho*	lambda supergroup	Sfi11-like[a]	3(II)	Yes (phage)	*S. epidermidis*	[36]
PH15	44,047	*Sipho*	lambda supergroup	Sfi11-like[a]	3(II)	Yes (phage)	*S. epidermidis*	[36]
phi909	30,124	*Sipho*	lambda supergroup	Sfi21-like[b]	ND	Yes (prophage)	*S. epidermidis FRI909*	[37]
vB_SepiS-phiIPLA5	43,581	*Sipho*	lambda supergroup	Sfi11-like[a]	ND	Yes (phage)	*S. epidermidis*	[38]
vB_SepiS-phiIPLA7	42,123	*Sipho*	lambda supergroup	Sfi11-like[a]	ND	Yes (phage)	*S. epidermidis*	[38]
phiTM300	45,700	*Sipho*	lambda supergroup	Sfi21-like[b]	ND	yes (prophage)	*S. carnosus TM300*	[39]
StB12	44,714	*Sipho*	lambda supergroup	Sfi11-like[a]	1(II)	yes (phage)	*S. hominis*	[40]
StB27	40,071	*Sipho*	lambda supergroup	Sfi11-like[a]	1(II)	yes (phage)	*S. hominis*	[40]
StB20	40,917	*Sipho*	lambda supergroup	Sfi21-like[a]	9(II)	yes (phage)	*S. capitis*	[40]

Prophages identified by genome strain sequencing and not further characterized are not indicated. (a) Based on Authors information, experimentally tested or determined on the basis of head genes pattern. (b) Determined on the basis of DNA packaging and head morphogenesis gene annotations during this analysis. (c) Following the classification proposed by [40].
Sipho: *Siphoviridae*; Ref, reference; ND, not determined.

References

1. Kwan, T.; Liu, J.; DuBow, M.; Gros, P.; Pelletier, J., The complete genomes and proteomes of 27 Staphylococcus aureus bacteriophages. *Proceedings of the National Academy of Sciences of the United States of America* **2005,** 102, (14), 5174-9.

2. Kaneko, J.; Kimura, T.; Narita, S.; Tomita, T.; Kamio, Y., Complete nucleotide sequence and molecular characterization of the temperate staphylococcal bacteriophage phiPVL carrying Panton-Valentine leukocidin genes. *Gene* **1998,** 215, (1), 57-67.
3. Zhang, M.; Ito, T.; Li, S.; Jin, J.; Takeuchi, F.; Lauderdale, T. L.; Higashide, M.; Hiramatsu, K., Identification of the third type of PVL phage in ST59 methicillin-resistant Staphylococcus aureus (MRSA) strains. *FEMS microbiology letters* **2011,** 323, (1), 20-8.
4. Narita, S.; Kaneko, J.; Chiba, J.; Piemont, Y.; Jarraud, S.; Etienne, J.; Kamio, Y., Phage conversion of Panton-Valentine leukocidin in Staphylococcus aureus: molecular analysis of a PVL-converting phage, phiSLT. *Gene* **2001,** 268, (1-2), 195-206.
5. Zou, D.; Kaneko, J.; Narita, S.; Kamio, Y., Prophage, phiPV83-pro, carrying panton-valentine leukocidin genes, on the Staphylococcus aureus P83 chromosome: comparative analysis of the genome structures of phiPV83-pro, phiPVL, phi11, and other phages. *Bioscience, biotechnology, and biochemistry* **2000,** 64, (12), 2631-43.
6. Ma, X. X.; Ito, T.; Chongtrakool, P.; Hiramatsu, K., Predominance of clones carrying Panton-Valentine leukocidin genes among methicillin-resistant Staphylococcus aureus strains isolated in Japanese hospitals from 1979 to 1985. *Journal of clinical microbiology* **2006,** 44, (12), 4515-27.
7. Baba, T.; Bae, T.; Schneewind, O.; Takeuchi, F.; Hiramatsu, K., Genome sequence of Staphylococcus aureus strain Newman and comparative analysis of staphylococcal genomes: polymorphism and evolution of two major pathogenicity islands. *Journal of bacteriology* **2008,** 190, (1), 300-10.
8. Ma, X. X.; Ito, T.; Kondo, Y.; Cho, M.; Yoshizawa, Y.; Kaneko, J.; Katai, A.; Higashiide, M.; Li, S.; Hiramatsu, K., Two different Panton-Valentine leukocidin phage lineages predominate in Japan. *Journal of clinical microbiology* **2008,** 46, (10), 3246-58.
9. Diep, B. A.; Gill, S. R.; Chang, R. F.; Phan, T. H.; Chen, J. H.; Davidson, M. G.; Lin, F.; Lin, J.; Carleton, H. A.; Mongodin, E. F.; Sensabaugh, G. F.; Perdreau-Remington, F., Complete genome sequence of USA300, an epidemic clone of community-acquired meticillin-resistant Staphylococcus aureus. *Lancet* **2006,** 367, (9512), 731-9.
10. Yamaguchi, T.; Hayashi, T.; Takami, H.; Nakasone, K.; Ohnishi, M.; Nakayama, K.; Yamada, S.; Komatsuzawa, H.; Sugai, M., Phage conversion of exfoliative toxin A production in Staphylococcus aureus. *Molecular microbiology* **2000,** 38, (4), 694-705.
11. Kuroda, M.; Ohta, T.; Uchiyama, I.; Baba, T.; Yuzawa, H.; Kobayashi, I.; Cui, L.; Oguchi, A.; Aoki, K.; Nagai, Y.; Lian, J.; Ito, T.; Kanamori, M.; Matsumaru, H.; Maruyama, A.; Murakami, H.; Hosoyama, A.; Mizutani-Ui, Y.; Takahashi, N. K.; Sawano, T.; Inoue, R.; Kaito, C.; Sekimizu, K.; Hirakawa, H.; Kuhara, S.; Goto, S.; Yabuzaki, J.; Kanehisa, M.; Yamashita, A.; Oshima, K.; Furuya, K.; Yoshino, C.; Shiba, T.; Hattori, M.; Ogasawara, N.; Hayashi, H.; Hiramatsu, K.; Whole genome

sequencing of meticillin-resistant Staphylococcus aureus. *Lancet* **2001,** 357, (9264), 1225-40.

12. Canchaya, C.; Proux, C.; Fournous, G.; Bruttin, A.; Brussow, H. Prophage Genomics. *Microbiology and Molecular Biology Reviews* **2003,** 67, (2), 238-276.
13. Sumby, P.; Waldor, M. K., Transcription of the toxin genes present within the Staphylococcal phage phiSa3ms is intimately linked with the phage's life cycle. *Journal of bacteriology* **2003,** 185, (23), 6841-51.
14. Gillaspy, A. F.; Worrell,V.; Orvis, J.; Roe, B.A.; Dyer, D.W.; Iandolo, J. J. The Staphylococcus aureus NCTC8325 Genome. In *Gram positive pathogens*, Fischetti, V., Novick,R., Ferretti,J., Portnoy,D. and Rood,J., Ed. ASM press: 2006.
15. Feng, Y.; Chen, C. J.; Su, L. H.; Hu, S.; Yu, J.; Chiu, C. H., Evolution and pathogenesis of Staphylococcus aureus: lessons learned from genotyping and comparative genomics. *FEMS Microbiol Rev* **2008,** 32, (1), 23-37.
16. Iandolo, J. J.; Worrell, V.; Groicher, K. H.; Qian, Y.; Tian, R.; Kenton, S.; Dorman, A.; Ji, H.; Lin, S.; Loh, P.; Qi, S.; Zhu, H.; Roe, B. A., Comparative analysis of the genomes of the temperate bacteriophages phi 11, phi 12 and phi 13 of Staphylococcus aureus 8325. *Gene* **2002,** 289, (1-2), 109-18.
17. Lee, C. Y.; Iandolo, J. J., Lysogenic conversion of staphylococcal lipase is caused by insertion of the bacteriophage L54a genome into the lipase structural gene. *Journal of bacteriology* **1986,** 166, (2), 385-91.
18. Bae, T.; Baba, T.; Hiramatsu, K.; Schneewind, O., Prophages of Staphylococcus aureus Newman and their contribution to virulence. *Molecular microbiology* **2006,** 62, (4), 1035-47.
19. Christie, G. E.; Matthews, A. M.; King, D. G.; Lane, K. D.; Olivarez, N. P.; Tallent, S. M.; Gill, S. R.; Novick, R. P., The complete genomes of Staphylococcus aureus bacteriophages 80 and 80alpha--implications for the specificity of SaPI mobilization. *Virology* **2010,** 407, (2), 381-90.
20. Matsuzaki, S.; Yasuda, M.; Nishikawa, H.; Kuroda, M.; Ujihara, T.; Shuin, T.; Shen, Y.; Jin, Z.; Fujimoto, S.; Nasimuzzaman, M. D.; Wakiguchi, H.; Sugihara, S.; Sugiura, T.; Koda, S.; Muraoka, A.; Imai, S., Experimental protection of mice against lethal Staphylococcus aureus infection by novel bacteriophage phi MR11. *The Journal of infectious diseases* **2003,** 187, (4), 613-24.
21. Hoshiba, H.; Uchiyama, J.; Kato, S.; Ujihara, T.; Muraoka, A.; Daibata, M.; Wakiguchi, H.; Matsuzaki, S., Isolation and characterization of a novel Staphylococcus aureus bacteriophage, phiMR25, and its therapeutic potential. *Archives of virology* **2010,** 155, (4), 545-52.
22. Garcia, P.; Martinez, B.; Obeso, J. M.; Lavigne, R.; Lurz, R.; Rodriguez, A., Functional genomic analysis of two Staphylococcus aureus phages isolated from the dairy environment. *Applied and environmental microbiology* **2009,** 75, (24), 7663-73.
23. Lee, Y. D.; Chang, H. I.; Park, J. H., Genomic sequence of temperate phage TEM126 isolated from wild type S. aureus. *Archives of virology* **2011,** 156, (4), 717-20.

24. Kim, M. S.; Myung, H., Complete Genome of Staphylococcus aureus Phage SA11. *Journal of virology* **2012,** 86, (18), 10232.
25. Vybiral, D.; Takac, M.; Loessner, M.; Witte, A.; von Ahsen, U.; Blasi, U., Complete nucleotide sequence and molecular characterization of two lytic Staphylococcus aureus phages: 44AHJD and P68. *FEMS microbiology letters* **2003,** 219, (2), 275-83.
26. Son, J. S.; Lee, S. J.; Jun, S. Y.; Yoon, S. J.; Kang, S. H.; Paik, H. R.; Kang, J. O.; Choi, Y. J., Antibacterial and biofilm removal activity of a podoviridae Staphylococcus aureus bacteriophage SAP-2 and a derived recombinant cell-wall-degrading enzyme. *Applied microbiology and biotechnology* **2010,** 86, (5), 1439-49.
27. O'Flaherty, S.; Coffey, A.; Edwards, R.; Meaney, W.; Fitzgerald, G. F.; Ross, R. P., Genome of Staphylococcal Phage K: a New Lineage of Myoviridae Infecting Gram-Positive Bacteria with a Low G+C Content. *Journal of bacteriology* **2004,** 186, (9), 2862-2871.
28. Son, J. S.; Kim, E. B.; Lee, S. J.; Jun, S. Y.; Yoon, S. J.; Kang, S. H.; Choi, Y. J., Characterization of Staphylococcus aureus derived from bovine mastitis and isolation of two lytic bacteriophages. *The Journal of general and applied microbiology* **2010,** 56, (4), 347-53.
29. Kwiatek, M.; Parasion, S.; Mizak, L.; Gryko, R.; Bartoszcze, M.; Kocik, J., Characterization of a bacteriophage, isolated from a cow with mastitis, that is lytic against Staphylococcus aureus strains. *Archives of virology* **2012,** 157, (2), 225-34.
30. Lobocka, M.; Hejnowicz, M. S.; Dabrowski, K.; Gozdek, A.; Kosakowski, J.; Witkowska, M.; Ulatowska, M. I.; Weber-Dabrowska, B.; Kwiatek, M.; Parasion, S.; Gawor, J.; Kosowska, H.; Glowacka, A., Genomics of staphylococcal Twort-like phages--potential therapeutics of the post-antibiotic era. *Advances in virus research* **2012,** 83, 143-216.
31. Synnott, A. J.; Kuang, Y.; Kurimoto, M.; Yamamichi, K.; Iwano, H.; Tanji, Y., Isolation from sewage influent and characterization of novel Staphylococcus aureus bacteriophages with wide host ranges and potent lytic capabilities. *Applied and environmental microbiology* **2009,** 75, (13), 4483-90.
32. Hsieh, S. E.; Lo, H. H.; Chen, S. T.; Lee, M. C.; Tseng, Y. H., Wide host range and strong lytic activity of Staphylococcus aureus lytic phage Stau2. *Applied and environmental microbiology* **2011,** 77, (3), 756-61.
33. Gu, J.; Liu, X.; Lu, R.; Li, Y.; Song, J.; Lei, L.; Sun, C.; Feng, X.; Du, C.; Yu, H.; Yang, Y.; Han, W., Complete genome sequence of Staphylococcus aureus bacteriophage GH15. *Journal of virology* **2012,** 86, (16), 8914-5.
34. Vandersteegen, K.; Mattheus, W.; Ceyssens, P. J.; Bilocq, F.; De Vos, D.; Pirnay, J. P.; Noben, J. P.; Merabishvili, M.; Lipinska, U.; Hermans, K.; Lavigne, R., Microbiological and molecular assessment of bacteriophage ISP for the control of Staphylococcus aureus. *PloS one* **2011,** 6, (9), e24418.
35. Kvachadze, L.; Balarjishvili, N.; Meskhi, T.; Tevdoradze, E.; Skhirtladze, N.; Pataridze, T.; Adamia, R.; Topuria, T.; Kutter, E.; Rohde, C.; Kutateladze, M.,

Evaluation of lytic activity of staphylococcal bacteriophage Sb-1 against freshly isolated clinical pathogens. *Microbial biotechnology* **2011,** 4, (5), 643-50.

36. Daniel, A.; Bonnen, P. E.; Fischetti, V. A., First complete genome sequence of two Staphylococcus epidermidis bacteriophages. *Journal of bacteriology* **2007,** 189, (5), 2086-100.

37. Madhusoodanan, J.; Seo, K. S.; Remortel, B.; Park, J. Y.; Hwang, S. Y.; Fox, L. K.; Park, Y. H.; Deobald, C. F.; Wang, D.; Liu, S.; Daugherty, S. C.; Gill, A. L.; Bohach, G. A.; Gill, S. R., An Enterotoxin-Bearing Pathogenicity Island in Staphylococcus epidermidis. *Journal of bacteriology* **2011,** 193, (8), 1854-62.

38. Gutierrez, D.; Martinez, B.; Rodriguez, A.; Garcia, P., Genomic characterization of two Staphylococcus epidermidis bacteriophages with anti-biofilm potential. *BMC genomics* **2012,** 13, (1), 228.

39. Rosenstein, R.; Nerz, C.; Biswas, L.; Resch, A.; Raddatz, G.; Schuster, S. C.; Gotz, F., Genome analysis of the meat starter culture bacterium Staphylococcus carnosus TM300. *Applied and environmental microbiology* **2009,** 75, (3), 811-22.

40. Deghorain, M.; Bobay, L. M.; Smeesters, P. R.; Bousbata, S.; Vermeersch, M.; Perez-Morga, D.; Dreze, P. A.; Rocha, E. P.; Touchon, M.; Van Melderen, L., Characterization of novel phages isolated in coagulase-negative Staphylococci reveals evolutionary relationships with S. aureus phages. *Journal of bacteriology* **2012,** 194, (21), 5829-39.
